# Supplementary material for: Impact of Intercurrent Introduction of Steroids on Clinical Outcomes in Advanced Non-Small-Cell Lung Cancer (NSCLC) Patients under Immune-Checkpoint Inhibitors (ICI)
Source: Cancers (Basel). 2020 Sep 30;12(10):2827. doi: 10.3390/cancers12102827 (PMC7599488; doi:10.3390/cancers12102827)
Supplement: Supplementary file 1 [file cancers-12-02827-s001.pdf]

cancers
MDPI

Supplementary Materials: Impact of Intercurrent Introduction of Steroids on Clinical Outcomes in Advanced Non-Small-Cell Lung Cancer (NSCLC) Patients under Immune-Checkpoint Inhibitors (ICI)

Andrea De Giglio, Laura Mezquita, Edouard Auclin, Félix Blanc-Durand, Mariona Riudavets, Caroline Caramella, Gala Martinez, Jose Carlos Benitez, Patricia Martín-Romano, Lamiae El-Amarti, Lizza Hendriks, Roberto Ferrara, Charles Naltet, Pernelle Lavaud, Anas Gazzah, Julien Adam, David Planchard, Nathalie Chaput and Benjamin Besse

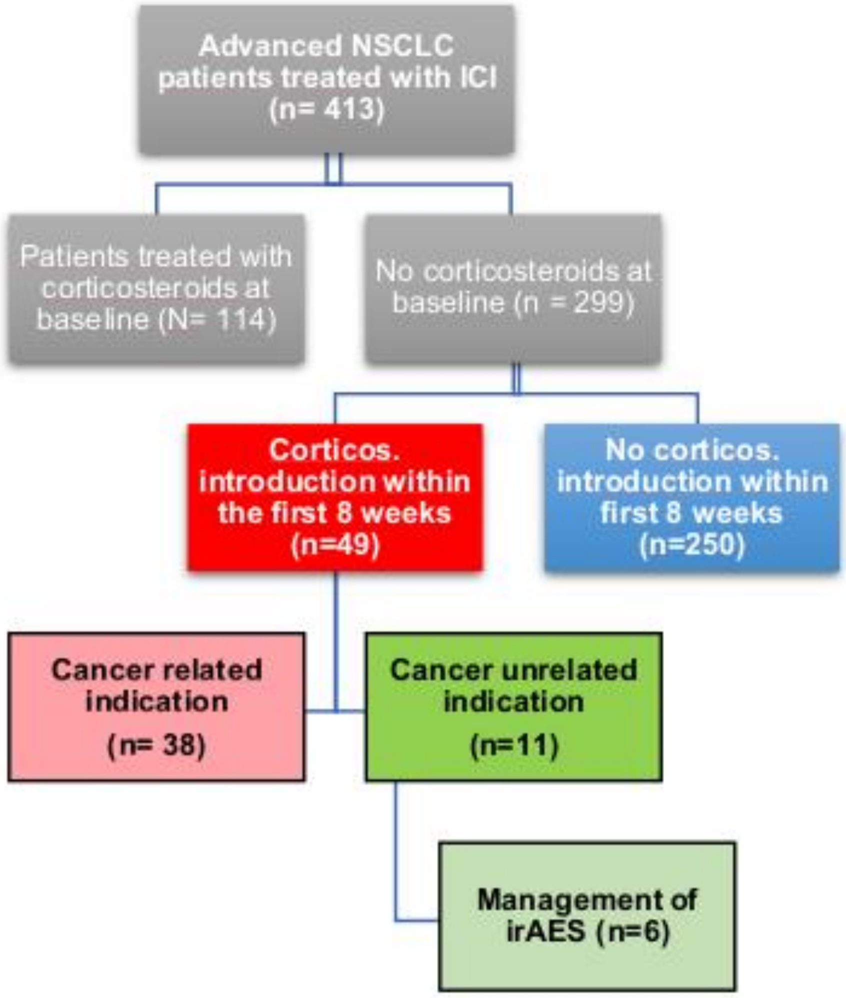

Figure S1: Flowchart of the study.

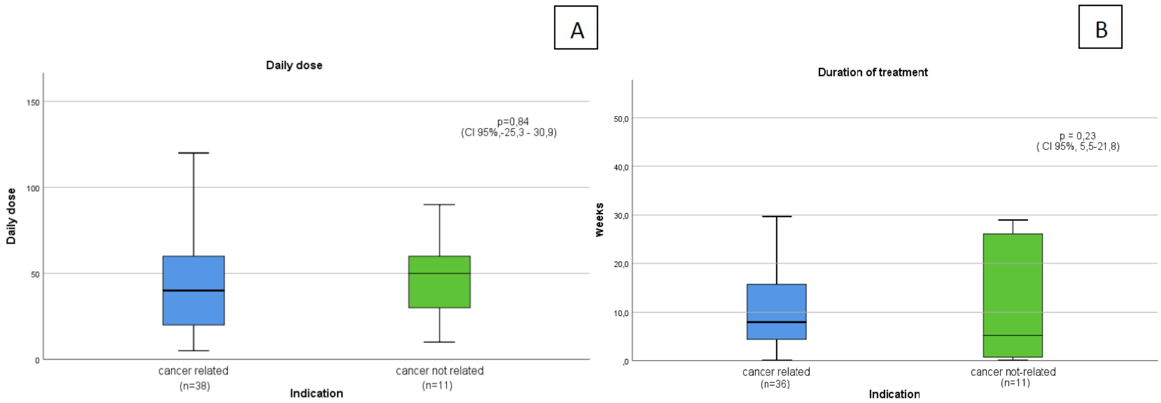

Figure S2. Box and whiskers plot describing daily dose (**A**) and duration of steroid treatment (**B**) per indication.

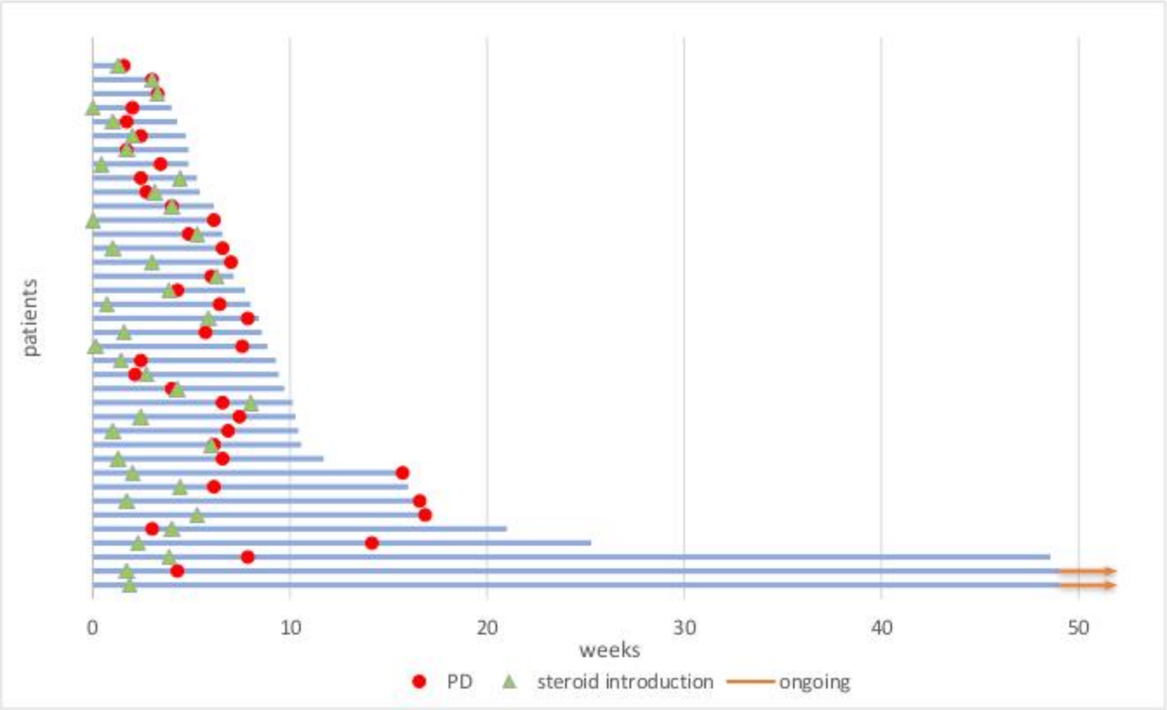

Figure S3: Swimmers plot detailing progression survival (PFS) and overall survival (OS) after 1 year of follow-up of patients receiving intercurrent steroids for cancer-related indications.

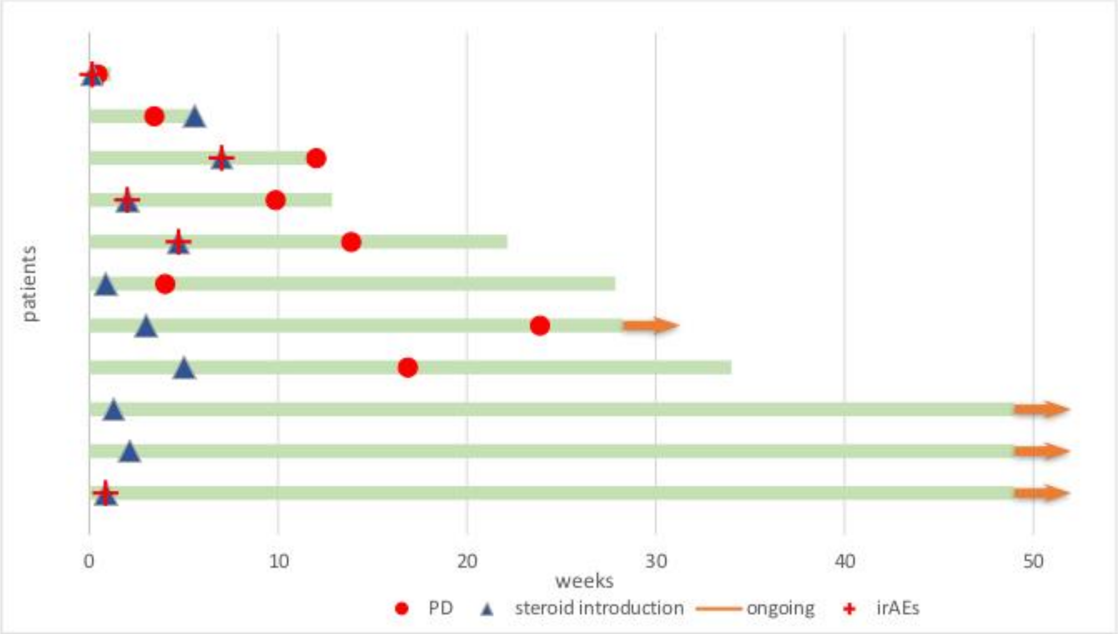

Figure S4: Swimmers plot detailing progression survival (PFS) and overall survival (OS) after 1 year of follow-up of patients receiving intercurrent steroids for cancer-unrelated indications.

Table S1: Cancer-related indications for corticosteroids administrations are listed with cumulative dosages.

Cancer-related indication	Number of patients	%
dyspnea cancer related	19	50
brain metastasis	6	15.8
tumor pain	3	7.9
sup. vena cava syndrome	3	7.9
Fatigue	2	5.3
Spinal compression	2	5.3
malignant pericarditis	1	2.6
Emesis/cholestasis/dysphagy	1	2.6
oncologic fever	1	2.6
Overall	38	100

Cancer-related indications for steroids administrations are listed with cumulative dosages.

Table S2: Non-cancer-related indications.

Non cancer-related indication	Number of patients	%
**Pneumonia/COPD exac.**	3	27.2
**Pneumonitis ir**	3	27.2
**Arthritis ir**	2	18.3
**Liver failure ir**	1	9.1
**Infusion reaction**	1	9.1
**Premedication for cementoplasty**	1	9.1
**Overall**	11	100

Non-cancer-related indications for steroids administrations are listed with cumulative dosages. Abbreviation: COPD, chronic obstructive pulmonary disease.

Table S3. Baseline Characteristics of study population according to antibiotic prescription.

		Whole sample	antibiotic no	antibiotic yes	p-value
		(N=413)	(N=265)	(N=148)	
**Steroid group**	baseline steroids	114 (28%)	62 (23%)	52 (35%)	0.002
	intercurrent steroids	49 (12%)	26 (10%)	23 (16%)	
	no steroids	250 (60%)	177 (67%)	73 (49%)	
**Reason of intercurrent prescription**	no steroids	250 (83%)	177 (87%)	73 (76%)	0.039
	non-cancer related	11 (4%)	5 (3%)	6 (6%)	
	cancer related	38 (13%)	21 (10%)	17 (18%)	
	NA	114	62	52	
**ECOG PS**	0-1	313 (77%)	221 (84%)	92 (64%)	<0.001
	≥ 2	95 (23%)	43 (16%)	52 (36%)	
	NA	5	1	4	

Abbreviation: ECOG PS, Eastern Cooperative Oncology Group performance status

© 2020 by the authors. Licensee MDPI, Basel, Switzerland. This article is an open access article distributed under the terms and conditions of the Creative Commons Attribution (CC BY) license (http://creativecommons.org/licenses/by/4.0/).
